# The Systemic Zinc Homeostasis Was Modulated in Broilers Challenged by *Salmonella*

**DOI:** 10.1007/s12011-019-01921-1

**Published:** 2019-10-22

**Authors:** Aimin Wu, Shiping Bai, Xuemei Ding, Jianping Wang, Qiufeng Zeng, Huanwei Peng, Bing Wu, Keying Zhang

**Affiliations:** 1 Institute of Animal Nutrition, Sichuan Agricultural University, Chengdu, 611130 Sichuan China; 2 Key Laboratory of Animal Disease-Resistance Nutrition, Ministry of Education, Chengdu, 611130 Sichuan China; 3 Sichuan Chelota Group, Liangshui Village, Jinyu Town, Guanghan City, 618300 Sichuan China; 4 Animal Nutrition Institute, Key Laboratory of Animal Disease-Resistance Nutrition, Ministry of Education, Sichuan Agricultural University, Huimin Road 211, Chengdu, 611130 China

**Keywords:** *Salmonella*, Broiler, Zinc, Hypozincaemia, Zinc homeostasis

## Abstract

*Salmonella* challenge leads to systemic responses that induce the hypozincaemia in mice, which is considered a vital strategy against *Salmonella* invasion. However, it is not yet known if this phenomenon occurs in broilers. To investigate the change in zinc homeostasis of broilers against *Salmonella* challenge, 1-day-old male broilers were fed with the basal diet for 7 days. Afterwards, broilers were orally inoculated with either 0 or 0.5 × 10^8^ CFU *Salmonella* Typhimurium (ST). The serum and selected tissues of *Salmonella*-challenged and non-challenged broilers were collected at 1, 3 and 7 days post-challenge for zinc homeostasis analysis. Our results showed that *Salmonella* challenge results in hypozincaemia (serum zinc decrease and liver zinc increase) via modulating the systemic zinc homeostasis of broilers. A profound, zinc transporter–mediated zinc absorption and redistribution affecting zinc homeostasis provided a mechanistic explanation for this phenomenon. In addition, we found that the zinc importers *Zip5*, *Zip10*, *Zip11*, *Zip12*, *Zip13* and *Zip14* were mainly downregulated in *Salmonella*-challenged broilers to reduce zinc absorption in the duodenum, while the *Zip14* mRNA expression was upregulated to redistribute zinc into the liver. Collectively, these findings reveal that broilers counteract *Salmonella* infection via modulating their systemic zinc homeostasis.

**Electronic supplementary material:**

The online version of this article (10.1007/s12011-019-01921-1) contains supplementary material, which is available to authorized users.

## Introduction

Food-borne *Salmonella* remains a major public health concern worldwide, being responsible for hundreds of millions of cases of human gastroenteritis [1–3]. Broiler meat contaminated with *Salmonella* is the primary vehicles for human salmonellosis [4, 5]. Aside from its impact on human health, *Salmonella* infection results in growth depression, intestinal inflammation, high mortality and cross-contamination in broilers [1, 6], which causes substantial economic loss to the poultry industry per year.

In mice, *Salmonella* infection induces hypoferraemia (serum iron decrease and liver iron increase), as iron plays a role in the regulation of the inflammatory response [7, 8]. Since other critical physiological functions also involve iron, all living organisms require iron to survive, including *Salmonella* [9]. The functions of hypoferraemia are considered to be the host defensive, because it decreases the availability of iron for *Salmonella* in a process termed “nutritional immunity” [10–13]. Besides iron, zinc also plays vital roles in host nutritional immunity [14, 15]. Similarly, hypozincaemia has also been observed after acute administration of numerous pathogens and agents, such as *Mycobacterium tuberculosis*, IL-6 and LPS [16–18]*.* This process is accompanied by a decrease in the serum zinc concentration and an increase in the zinc content in the liver due to the altered activity of zinc transporters, especially upregulation of *Zip14* gene expression [16]. Meanwhile, there is an increased expression of zinc-binding protein metallothionein (MT) via a mechanism associated with oxidative stress [16, 19]. Notably, enhancing MT expression availability controls the “free zinc” (labile zinc that is available for binding by newly synthesized zinc metalloproteins) concentration in cells, and limits *Salmonella* infection in macrophages [20]. Thus, hypozincaemia has been considered an effective strategy to limit pathogens from acquiring sufficient zinc for infection and proliferation in mice [16]. Interestingly, a similar phenotype was also observed in broilers under *Escherichia coli* or LPS stimulation [21]. In this context, broilers could also use hypozincaemia as a useful defence strategy against pathogen infection. However, this has not yet been studied in broilers under *Salmonella* infection, and the roles and mechanisms of hypozincaemia in broilers are also largely unknown.

Therefore, in this study, we investigated the impact of *Salmonella* challenge on the systemic zinc homeostasis of broilers and revealed how broilers modulate their zinc homeostasis to counteract *Salmonella* infection.

## Materials and Methods

### Animals and Diets

A total of 48, 1-day-old Arbor Acres (AA) male broilers were fed the basal diet for 7 days. Afterwards, the broilers were randomly divided into two treatment groups: non-challenged control group; *Salmonella-*challenged group. The basal diet (Table 1) was formulated to meet the requirements recommended by the National Research Council. All broilers were placed in a single thermo-controlled room. Room temperature was maintained at 32 °C during the first 3 days of life and then decreased by 2 to 3 °C per week. Broilers were given ad libitum access to feed and water and 24-h illumination throughout the whole experimental trial. The experimental procedures used in this study were approved by the Animal Care Advisory Committee of Sichuan Agricultural University.

**Table 1:** Composition and nutrient concentrations of the diet (air dry-basis, %)

Ingredients	Amount	Calculated nutrient concentrations	Amount
Corn	54.30	Metabolisable energy (kcal/kg)	2950.00
Soybean meal	38.12	Crude protein	21.00
Soybean oil	3.40	Calcium	1.01
L-Lysine hydrochloride	0.15	Non-phytate phosphorus	0.45
DL-Methionine	0.25	Lysine	1.15
Calcium carbonate	1.14	Methionine	0.50
Dicalcium phosphate	1.86	Methionine and cystine	0.86
Sodium chloride	0.40		
Choline chloride	0.15		
Premix^a^	0.23		

^a^Supplied the following per kilogram of complete feed: Cu (CuSO_4_·5H_2_O), 8 mg; Fe (FeSO_4_·7H_2_O), 100 mg; Mn (MnSO_4_·7H_2_O), 120 mg; Zn (ZnSO_4_·7H_2_O), 80 mg; Se (Na_2_SeO_3_), 0.3 mg; I (KI), 0.70 mg; vitamin A (retinyl palmitate), 8000 IU; cholecalciferol, 1000 IU; vitamin E (DL-tocopheryl acetate), 20 IU; thiamine, 0.8 mg; riboflavin, 2.5 mg; pyridoxine, 1.5 mg; pantothenic acid, 2.2 mg; folic acid, 0.55 mg; nicotinic acid, 35 mg; and biotin, 0.18 mg

### Oral *Salmonella* Inoculation

On day 7, broilers were orally inoculated with either 0 or 0.5 × 10^8^ CFU *Salmonella enterica serovar* Typhimurium (ST), according to the previous assignment (non-challenged vs. challenged). The method is detailed elsewhere [22]. The strain of ST used in this experiment was from the *American Type Culture Collection* (ATCC, No. 14028).

### Growth Performance

The body weight of broilers was recorded at 7, 8, 10 and 14 days of age. These values were used to calculate the average body weight gain, according to the body weight of each growth phase.

### Sample Collection and Procedures

At 1, 3 and 7 days post-challenge (at 8, 10 and 14 days of age), blood samples were taken from eight randomly selected birds in each group, and centrifuged at 2500 g/min for 10 min at 4 °C and then serum layer stored at − 20 °C for serum zinc concentration analysis. Afterwards, the broilers were sacrificed by CO_2_ to collect the liver, spleen, thymus, bursa of Fabricius, duodenum, jejunum, ileum and cecum for the determination of zinc content and expression levels of zinc metabolism-related genes. Note that selected birds had fasted 12 h before sample collection.

### RT-PCR

Total RNA was extracted from the liver and duodenum using RNAiso Plus reagent (TaKaRa), according to the manufacturer’s protocol and transcribed into cDNA by using the Prime Script™ RT reagent kit (TaKaRa). Quantitative real-time PCR system was performed on a CFX96 PCR system (BioRad) with the oligonucleotide sequences shown in Table S1. Relative gene expression was calculated with the 2^**△△**Ct^ method [23], normalizing the results to the house-keeping gene β-actin.

### Zinc Measured by Inductively Coupled Plasma Mass Spectroscopy

An Agilent 7500cx inductively coupled plasma mass spectroscopy (ICP-MS) instrument (G3148B ISIS, Agilent Technologies, Japan) equipped with a G3160B I-AS integrated autosampler was employed to measure the ion profile since it allows a reduction in the detection time and volume of each sample compared with similar instruments. The typical operating conditions and the pretreatments of samples used in this study have been described previously [24].

### Statistical Analysis

Statistical analysis was performed using GraphPad Prism software (Version 5.01). All results were presented as mean ± SEM. Statistical tests included the unpaired two-tailed Student’s *t* test as appropriate with Bonferroni post hoc tests. Significance (*P* value) was evaluated at the 0.05 level.

## Results

### *Salmonella* Challenge Decreased the Growth Performance of Broilers

**Fig. 1:**
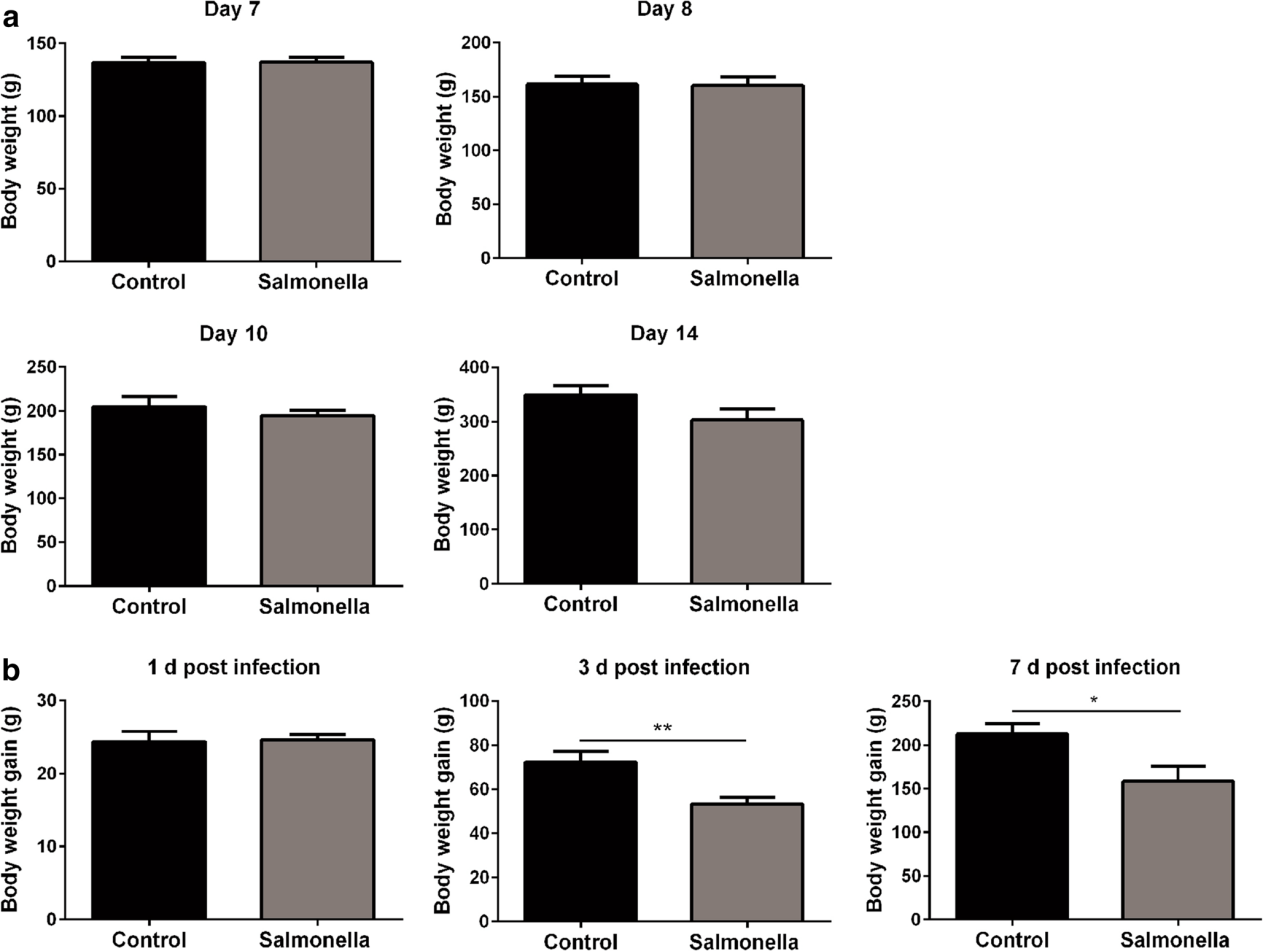
The growth performance of 7~14 day-old broilers. **a** The average body weight of broilers at 7, 8, 10 and 14 days of age. **b** The average body weight gain of broilers at 1, 3 and 7 days post-challenge (*n* = 8). *P < 0.05, **P < 0.01, ***P < 0.001, all data compare with control, the same with the follow figures

Our results showed that there was no significant difference in the average body weight between the control and *Salmonella*-challenged broilers (Fig. 1a), with only a slight tendency towards a decreased body weight of broilers at 14 days (*P* = 0.0953). In contrast, *Salmonella* challenge dramatically reduced the average body weight gain of broilers at 3 and 7 days post-challenge (Fig. 1b).

### Hypozincaemia Was Observed in *Salmonella*-Challenged Broilers

**Fig. 2:**
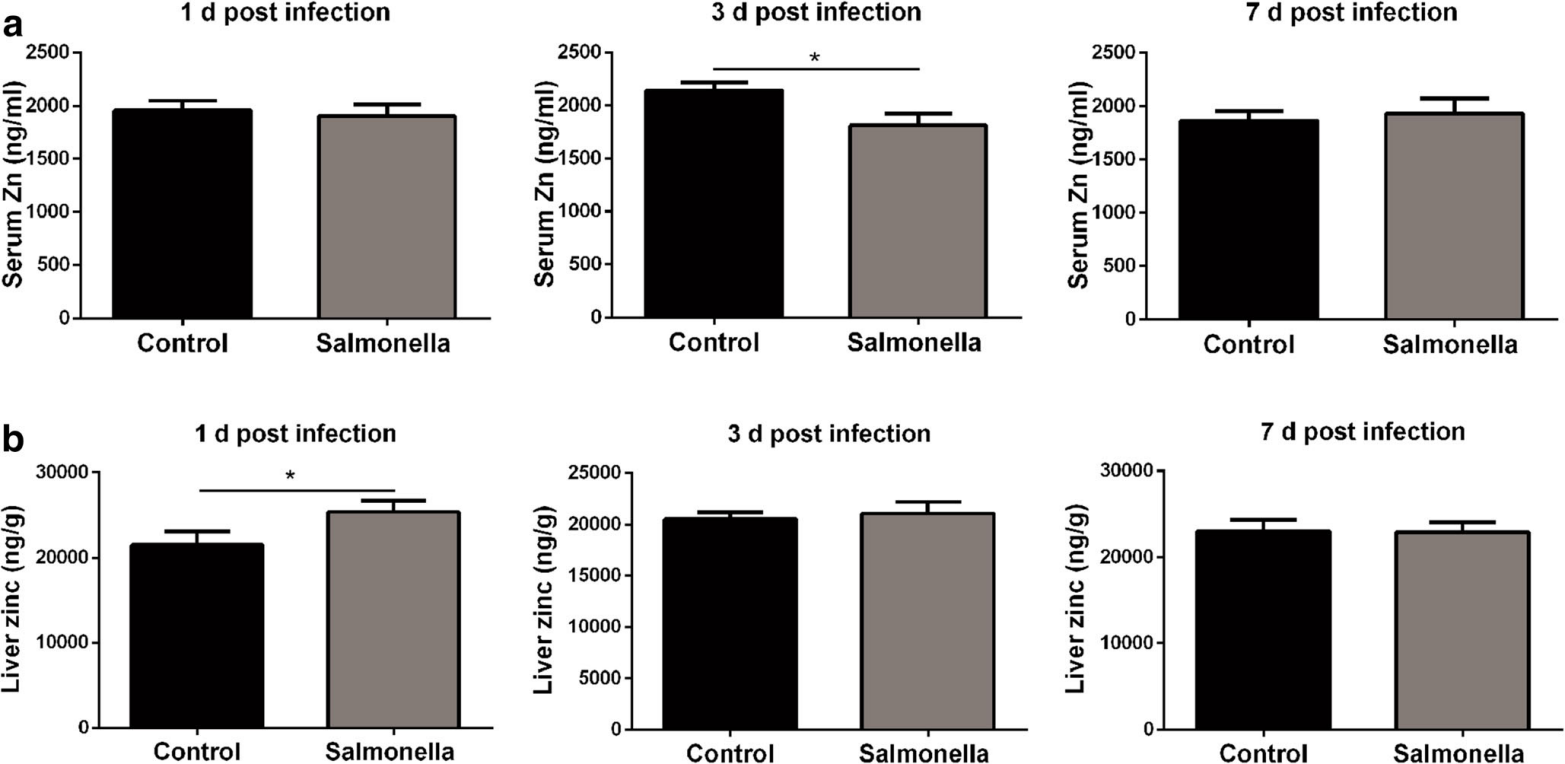
Hypozincaemia was observed in *Salmonella* challenged broilers. The serum zinc concentration (**a**) and liver zinc content (**b**) of broilers at 1, 3 and 7 days post-challenge were detected by ICP-MS (*n* = 8)

Generally, mice challenged with *Salmonella* display profound changes in their metal metabolism [25]. In the case of zinc, “hypozincaemia” is among the changes observed in the period of acute inflammatory response. It is considered an effective strategy for mice to combat *Salmonella* challenge [16]. In broilers, hypozincaemia was also observed following *Salmonella* challenge (Fig. 2). *Salmonella* challenge resulted in a serum zinc decrease at 3 days post-challenge (Fig. 2a) and a liver zinc content increase, zinc was redistributed into the liver at 1 day post-challenge (Fig. 2b).

### Zinc Was Also Redistributed into the Bursa of Fabricius

**Fig. 3:**
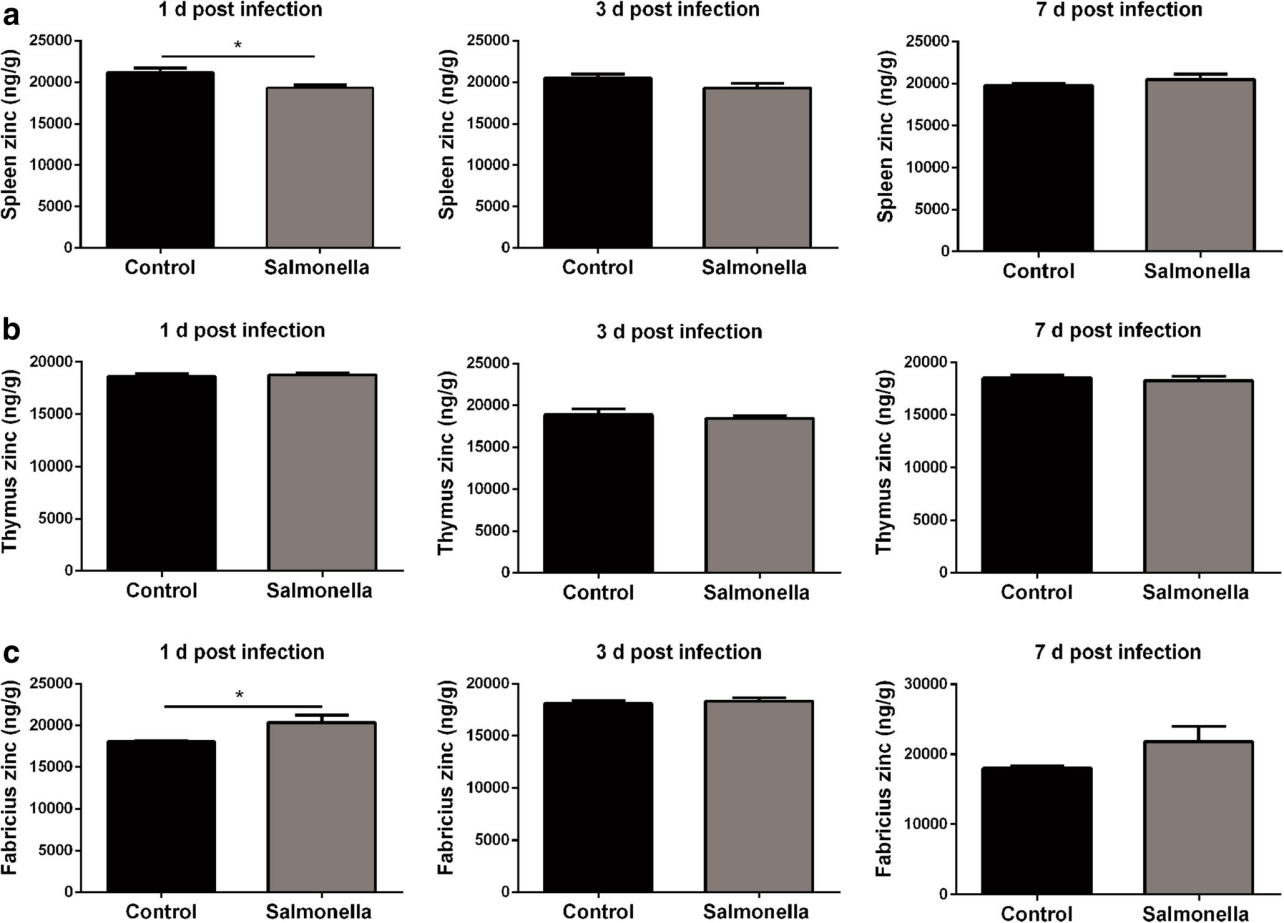
Zinc was redistributed into the bursa of Fabricius. Zinc content in the spleen (**a**), thymus (**b**) and bursa of Fabricius (**c**) zinc content of broilers at 1, 3 and 7 days post-challenge were detected by ICP-MS (*n* = 8)

As noted above, zinc was redistributed into the liver in *Salmonella*-challenged broilers (Fig. 2), which is considered to be a response by the host defence system. A great deal of literature has already revealed that immune organs play crucial roles in the defence against *Salmonella* [22]. Whether the host will alter the zinc metabolism of their immune organs in response to *Salmonella* challenge remains unknown until now. We checked the zinc content in three different immune organs of broilers. As shown in Fig. 3, *Salmonella* challenge altered the zinc metabolism in the spleen and bursa of Fabricius. However, there was no difference in the content of zinc in the thymus (Fig. 3b). Intriguingly, *Salmonella* challenge slightly reduced the zinc content in the spleen (Fig. 3a), but significantly increased the zinc content in the bursa of Fabricius, suggesting that zinc was also redistributed into the bursa of Fabricius in *Salmonella*-challenged broilers.

### *Salmonella* Challenge Inhibited the Zinc Absorption in the Intestine

**Fig. 4:**
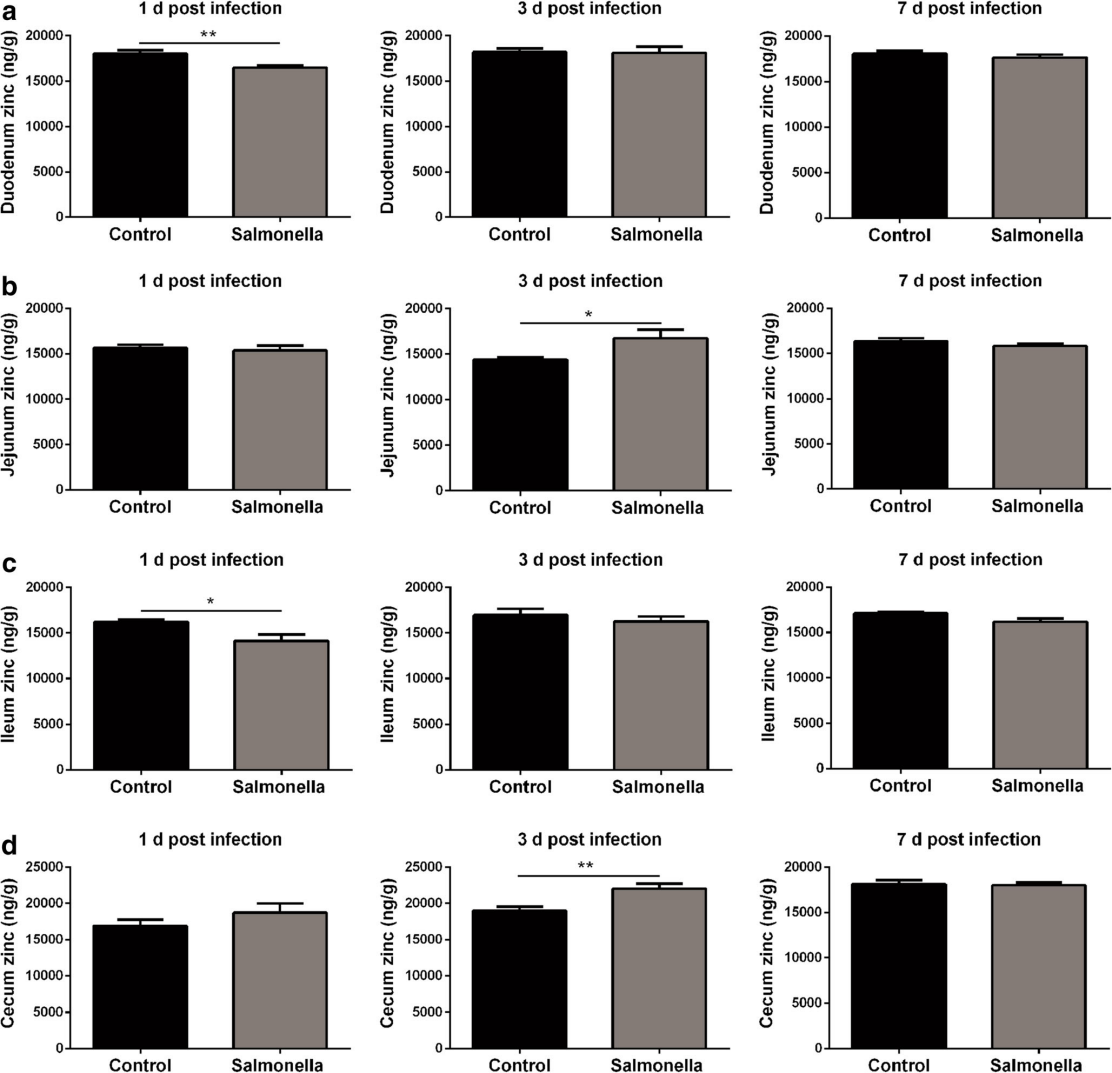
*Salmonella* challenge inhibited the zinc absorption in the small intestine. Duodenal (**a**), jejunal (**b**), ileal (**c**) and cecal (**d**) zinc content of broilers at 1, 3 and 7 days post-challenge were detected by ICP-MS (*n* = 8)

As zinc is not stored in body, it must to be ingested daily and its homeostasis must to be accurately regulated. Whether the host mediates the hypozincaemia against *Salmonella* challenge through limiting the absorption of zinc was not fully known. Therefore, the duodenal, jejunal, ileal and cecal contents of zinc were measured by ICP-MS in this study. As shown in Fig. 4, *Salmonella* challenge inhibited zinc absorption in the duodenum, and the zinc content of the duodenum in *Salmonella*-challenged broilers was less compared with the control group at the 1 day post-challenge (Fig. 4a). Similar results were also observed in the ileum (Fig. 4c). Interestingly, *Salmonella* challenge resulted in zinc accumulation in the jejunum and cecum at 3 days post-challenge (Fig. 4b, d).

### Zinc Transporter–Mediated Hypozincaemia in *Salmonella*-Challenged Broilers

**Fig. 5:**
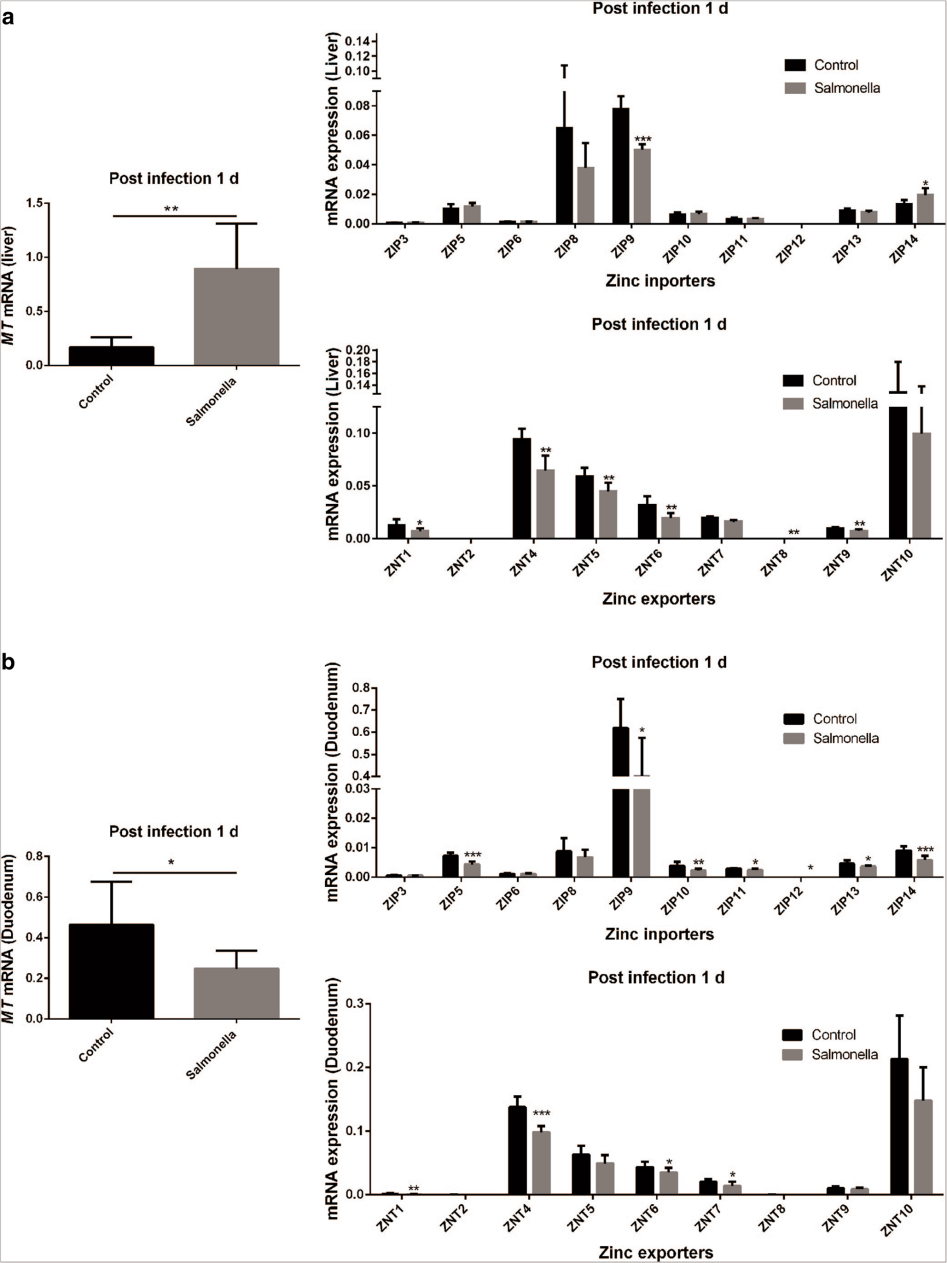
Zinc transporter–mediated zinc redistribution in *Salmonella*-challenged broilers. Zinc metabolism relative gene mRNA expression in liver (**a**) and duodenum (**b**) of broilers at 1 day post-challenge (*n* = 8)

Figures 2b and 4a showed that *Salmonella* challenge altered the zinc homeostasis of the liver and duodenum, which plays a crucial role in regulating the systemic zinc homeostasis. The *MT* mRNA expression was significantly upregulated in the liver at 1 day post-challenge (Fig. 5a). In addition, the host upregulated *Zip14* (a zinc importer) mRNA expression to accumulate zinc in the liver (Fig. 5a). On the contrary, the mRNA expressions of zinc exporters *ZnT1*, *ZnT4*, *ZnT5*, *ZnT6*, *ZnT8* and *ZnT9* in the liver were significantly downregulated in *Salmonella-*challenged broilers. Meanwhile, *Salmonella* challenge caused a significantly decrease in *MT* mTNA expression that was accompanied by differential expression of specific zinc transporters in the duodenum at 1 day post-challenge (Fig. 5b). The host limited the zinc absorption in the duodenum by downregulating the mRNA expression of zinc importers, such as *Zip5*, *Zip9*, *Zip10*, *Zip11*, *Zip12*, *Zip13* and *ZIP14*, and decreasing the mRNA expression levels of the zinc exporters *ZnT1*, *ZnT4*, *ZnT6* and *ZnT7* mRNA expression (Fig. 5b).

## Discussion

*Salmonella* challenge results in diarrhoea and severely reduces the body weight of animals [1, 26]. The impact of *Salmonella* challenge on the performance of broilers has been reported and was further confirmed in this study. *Salmonella* challenge substantially decreased the body weight gain of *Salmonella-*challenged broilers at 3 and 7 days post-challenge. *Salmonella* challenge impairs the intestinal mucosal barrier and affects the absorption, transfer and utilization of nutrients of the host, which explains this phenomenon [27, 28].

One of the most characteristic features of the acute-phase response to pathogen challenge is a dramatic change in the metabolism of ions, mainly transition metal ions, such as iron, zinc, copper and manganese [25], which are essential for host and pathogen. Consequently, the host has evolved sophisticated sequestration mechanisms to limit pathogen access to these ions [10]. These processes of host-enforced micronutrient restriction are termed “nutritional immunity” [10, 12]. Hypozincaemia induced by *Salmonella* challenge is believed to belong to the defence arsenal of nutritional immunity [16]. In line with the literature evidence, we revealed that *Salmonella*-challenged broilers also display hypozincaemia. Zinc was redistributed into the liver during the process of acute-phase response to *Salmonella* challenge, which was also confirmed by the *MT* mRNA expression in the liver. In addition, we found that the host upregulated zinc importer *Zip14* gene expression to redistribute zinc into the liver and downregulated the gene expression levels of zinc exporters (*ZnT4*, *ZnT5*, *ZnT6*, *ZnT8* and *ZnT9*) to accumulate zinc into the liver. Hypozincaemia is beneficial to reduce zinc availability for *Salmonella*, which limits *Salmonella* replication and formation of virulence gene formation [29, 30] and redistributes zinc into the liver for hepatic synthesis of acute-phase response proteins [16, 31]. Thus, it is not surprising that hypozincaemia is an important innate defence strategy.

Accumulating literature evidence shows that immune organs play crucial roles in defence against *Salmonella* infection [32–34], and mild zinc alteration dramatically affects the function of immune organs [35]. Therefore, we measured the zinc content in the spleen, thymus and bursa of Fabricius. As expected, *Salmonella* infection causes zinc redistribution into the bursa of Fabricius, a primary central humoral immune organ responsible for establishment and maintenance of the B cell compartment in avian species [36, 37]. Zinc accumulation contributes to B cell proliferation and enhances the immune function of the host to against bacterial infection. Furthermore, we also found substantial changes among other ions in the bursa of Fabricius of *Salmonella*-challenged broilers (data not shown). However, no remarkable changes in zinc were observed in the spleen and thymus.

As zinc is not stored in the body, it has to be ingested daily and its homeostasis needs to be regulated accurately. *Salmonella* challenge inhibited zinc absorption in the duodenum via downregulation of zinc importer (*Zip5*, *Zip10*, *Zip11*, *Zip12*, *Zip13* and *Zip14*) mRNA expression, which locate at membranes of cells in mammals and are responsible for zinc absorption from the gut tract. Notably, zinc exporters *ZnT1*, *ZnT4*, *ZnT6* and *ZnT7* were also downregulated in the duodenum of *Salmonella*-challenged broilers. These zinc exporters locate at the basement membrane or organelles in the duodenum and contribute to transport zinc from the intestinal epithelium to blood [38–41]. Hence, downregulation of these zinc exporters will significantly lessen the serum zinc concentration, which provides a mechanistic explanation for hypozincaemia.

Overall, we found that the systemic zinc homeostasis of broilers was modulated by *Salmonella*. *Salmonella* challenge induced hypozincaemia via limiting zinc absorption in the duodenum and redistributing zinc into the liver and bursa of Fabricius. Zinc transporters play a crucial role in this process, especially ZIP14. These changes in broilers seem to belong to the defence arsenal of the host.

## Electronic supplementary material

ESM 1 (DOCX 14 kb)
