# Supplementary material for: The Systemic Zinc Homeostasis Was Modulated in Broilers Challenged by Salmonella
Source: Biol Trace Elem Res. 2019 Oct 22;196(1):243–51. doi: 10.1007/s12011-019-01921-1 (PMC7289780; doi:10.1007/s12011-019-01921-1)
Supplement: Supplementary file 1 — (DOCX 14 kb) [file 12011_2019_1921_MOESM1_ESM.docx]

**Table S1 Sequences of primers used in real-time PCR assays**

| Gene name | Forward primer (5’-3’) | Reverse primer (5’-3’) |
| --- | --- | --- |
| *Actin* | GAGAAATTGTGCGTGACATCA | CCTGAACCTCTCATTGCCA |
| *MT* | ACTGTGCCAAGGGCTGTG | CATTTATTTCCTCCATCGGTAT |
| *Zip3* | AGCACAGCCGTCACTCCCA | AGCACTCTGCCTCCCTCCTC |
| *Zip5* | TGGTGGTGCTGGGAGACG | GGGGAGGCTGTGGGAGAA |
| *Zip6* | CCCAAATACCCAAGAGTG | TGTGGCAGACAGCAATAC |
| *Zip8* | TGTAAATGTCTCGGTGGG | CAAGATGGCTATGGAGGT |
| *Zip9* | GGAAGCATCACCCAGCAA | GCCAAGGACAAGCGAAAC |
| *Zip10* | CACAGTCACCTCTGGCATCA | CCTATCGCTAAGCCGTCA |
| *Zip11* | AGCCTTCGCTTTCCTCCC | ACTTCTCCTTCGCCACTCG |
| *Zip12* | GTAATCTGTGCCTTGAACC | CTTCCAAATCTCCACCAG |
| *Zip13* | TCTTGACTTTCTTGGCACTA | GCACTCCTCCTCTTCTTTC |
| *Zip14* | GTTCTGCCCCGCTGTCCT | GGTCTGCCCTCCTCCGTCT |
| *ZnT1* | CTTCGCTTAGCATTTCTT | TCTCCGATTTAGTCCTTCT |
| *ZnT2* | TCAGCCTCTTCGCCCTCT | GGTGCCAGCCAAAGTTCAT |
| *ZnT4* | CCGTCCTCTATCTGCTCTTCAT | AACCTCTTTGTGGGAGACTTTG |
| *ZnT5* | GAGGACCAGCCAAGACAA | ACCCAGGAAAGCAATGAC |
| *ZnT6* | CTTCCTGCTAATGTGGTG | ACTGGACTGGGTTTCTTC |
| *ZnT7* | TTCAGGAAGATGTCGGGATG | AGAAGAGGTTGCGGGAGG |
| *ZnT8* | GAGATTACAGGTGGGCAGAT | TAGGAGGCTTGGAGGTCA |
| *ZnT9* | TCAAGGGACCAGGGAAGG | CCCGTGTAAACCCAAGCA |
| *ZnT10* | TCTTGTCTTCGGCGTTCC | TAGCCTGTCAGTCAGTAGTTGC |
| *S100A9* | CGCATCATCCATCAAGAG | GACATCAATGGCTTTCTCC |
